# Discordance between levator hiatus morphology and function in pelvic floor dysfunction

**DOI:** 10.1097/MD.0000000000051318

**Published:** 2026-11-06

**Authors:** Yuhong Xue, Liang Mu, Shuliang Nan

**Affiliations:** a Department of Ultrasound Diagnosis, Shaanxi Provincial People’s Hospital, Xi’an, Shaanxi Province, China.

**Keywords:** bladder prolapse, levator hiatus, pelvic floor anatomy, pelvic floor function

## Abstract

This study aimed to characterize static anatomical and dynamic functional parameters of the levator hiatus in women with bladder prolapse and to examine their diagnostic performance. Primiparous women with a diagnosis of bladder prolapse were recruited as the prolapse group (n = 62), while healthy nulliparous women were included as the control group (n = 60). Nulliparous controls were selected to provide a reference group without childbirth-related pelvic floor remodeling; however, because parity differed systematically between groups, parity-related effects cannot be separated from prolapse-related effects in this case-control design. The levator hiatus area was measured both at rest and during maximal Valsalva maneuver, and the difference between these 2 measurements was calculated. Hierarchical regression analysis was performed to evaluate the independent and joint contributions of group status and body mass index (BMI) to these parameters. Receiver operating characteristic (ROC) curve analysis was used to assess diagnostic performance within the study cohort. The levator hiatus resting area, Valsalva area, and area change were all significantly greater in the prolapse group than in the control group (*P* < .001). Static anatomical parameters, including the resting and Valsalva areas, were associated with group status (*P* < .001), whereas the dynamic functional parameter, namely the area change, was associated with both group status (standardized β = 0.829, *P* < .001) and BMI (standardized β = 0.139, *P* = .003). ROC analysis showed very high apparent diagnostic performance for area change in this cohort (AUC = 1.000), compared with the static anatomical parameters (AUC = 0.999 and 0.920, respectively). These findings indicate different statistical association patterns for levator hiatus morphology and dynamic area change. Dynamic functional assessment may provide complementary information beyond static anatomical measures; however, its diagnostic performance requires confirmation in parity-matched and independent validation cohorts.

## 1. Introduction

Bladder prolapse is one of the most common pelvic floor dysfunction (PFD) disorders in postpartum women. As the most critical supportive structure of the pelvic floor, the levator ani muscle has drawn widespread attention regarding the role of its hiatus morphology in the development and progression of pelvic organ prolapse (POP).^[1]^ However, existing research has primarily focused on static anatomical measurements, leaving a gap in the understanding of dynamic pelvic floor function. The distensibility of the levator hiatus – its ability to change between rest and stress states – may capture a different aspect of pelvic floor behavior from a single static measurement. Yet, it remains unclear whether this dynamic feature provides information distinct from traditional morphological indicators and how it is associated with factors such as body mass index (BMI).^[2]^ In this study, the term “discordance” refers specifically to differing statistical association patterns between static morphological measures and dynamic area expansion, particularly with respect to BMI; it does not imply a patient-level mismatch or prove the existence of separate causal mechanisms. This study therefore aimed to compare static morphological and dynamic functional parameters of the levator hiatus in women with bladder prolapse and controls and to explore their associations with group status and BMI.

## 2. Materials and methods

### 2.1. Study population

This study was approved by the Ethics Committee of Shaanxi Provincial People’s Hospital. From July 2015 to June 2017, primiparous women diagnosed with bladder prolapse after puerperium at our hospital were enrolled in the prolapse group (n = 62), while healthy nulliparous women served as the control group (n = 60). Nulliparous women were selected as controls to provide a reference group without pregnancy- and childbirth-related pelvic floor remodeling and to maximize separation from postpartum pelvic floor abnormalities. This design choice also introduces an important limitation: parity is completely aligned with case-control status, so observed between-group differences may reflect a combination of bladder prolapse, pregnancy, and childbirth-related effects rather than prolapse alone. Exclusion criteria included a history of PFD prior to pregnancy, pelvic floor surgery or trauma, inflammation in the examined area, or inability to cooperate with the examination. All participants provided informed consent.

### 2.2. Methods

#### 2.2.1. Four-dimensional pelvic floor ultrasound

Four-Dimensional Pelvic Floor Ultrasound: A GE Voluson E8 ultrasound system equipped with RIC5-9-D and RAB4-8-D volume transducers was used. Participants were instructed to empty their rectum and bladder before examination. Scans were performed in the supine lithotomy position. Starting from the midsagittal plane, parameters were adjusted to obtain satisfactory two-dimensional images, followed by volume scanning. A suitable region of interest was selected, and 3D/4D acquisition was performed to dynamically observe pelvic floor structures and levator hiatus changes at rest and during maximum Valsalva maneuver. Operator-specific identifiers and blinding status were not available in the study records used for the present analysis; therefore, the exact number of examiners and whether ultrasound investigators were blinded to clinical diagnosis cannot be reliably reported. This lack of documentation is acknowledged as a potential source of measurement and observer bias.

#### 2.2.2. Measured parameters

Anatomical and functional changes of the pelvic floor were measured at rest and during the maximum Valsalva maneuver. The levator hiatus area was measured on 4D reconstructed images.

#### 2.2.3. Statistical analysis

Data were analyzed using SPSS 22.0. Continuous variables were expressed as mean ± standard deviation (x̄ ± S). Group comparisons were conducted using *t*-tests. Spearman rank correlation was used to analyze the relationship between BMI and static morphological indicators (resting area, Valsalva area) as well as the dynamic functional indicator (area expansion). Hierarchical regression analysis was applied to examine the independent and joint effects of group and BMI on levator hiatus parameters. The dependent variables were resting area, Valsalva area, and area expansion. Model improvement was assessed based on changes in R^2^ and their significance. Receiver operating characteristic (ROC) curve analysis was used to evaluate the diagnostic value of each indicator for POP, and the DeLong test was employed to compare differences in area under the curve (AUC). A *P*-value < .05 was considered statistically significant.

## 3. Results

1. The distribution of static and dynamic indicators of the levator hiatus in the prolapse and control groups is shown in Figure 1. Women in the prolapse group exhibited different types of bladder prolapse: 9 cases (15%) of Green type I, 39 cases (65%) of Green type II, and 12 cases (20%) of Green type III. There were no significant differences in age or height between the 2 groups (all *P* > .05). However, the prolapse group had significantly higher weight and BMI compared to the control group (all *P* < .05). The levator hiatus area at rest, during maximum Valsalva, and the area expansion were all significantly larger in the prolapse group (all *P* < .05, Table 1).
2. Correlation analysis between BMI and levator hiatus morphology and function showed that BMI was significantly positively correlated with both the static and dynamic functional indicators (ρ = 0.489, 0.611, and 0.657 for resting area, Valsalva area, and area expansion, respectively, all *P* < .05; Table 2).
3. To examine whether BMI influences group differences in levator hiatus area, hierarchical regression analysis was performed (Table 3). Model 1 included only the group variable. The BMI linear term (Model 2), BMI nonlinear term (Model 3), and the group-BMI interaction term (Model 4) were sequentially added. For static morphological indicators (resting area and Valsalva area), only the group contributed significantly (explaining 55.2 and 82.6% of the variance, respectively, *P* < .001). The changes in R^2^ for all subsequent models were not significant (all *P* > .05), indicating that BMI had no significant main or moderating effect on static levator hiatus morphology.
4. In contrast, the dynamic functional indicator (area expansion) was significantly influenced by both group and BMI. After adding BMI, the model’s explanatory power increased significantly from 83.9 to 85.0% (ΔR^2^ = 0.012, *P* = .003). Group was the strongest predictor of the dynamic functional indicator (B = 3.847, standardized β = 0.829, *P* < .001), indicating that after controlling for BMI, the area expansion in the prolapse group remained significantly higher than in the control group. BMI was also an independent significant predictor (B = 0.246, standardized β = 0.139, *P* = .003), suggesting that a higher BMI is associated with greater functional expansion of the levator hiatus (Table 4).
5. ROC curve analysis was used to evaluate the diagnostic performance of each indicator for POP within the study cohort (Fig. 2). The area expansion, reflecting dynamic change in the levator hiatus, showed an apparent AUC of 1.000 (95% CI: 1.000–1.000). At the data-derived optimal cutoff value of 5.85 cm^2^, sensitivity and specificity were both 100% in this cohort. The static morphological indicators (Valsalva area and resting area) also showed high apparent diagnostic performance (AUC = 0.999 and 0.920, respectively) (Table 5). Because the cutoff and performance estimates were derived and evaluated in the same case-control sample, these values should be interpreted as internal, exploratory estimates rather than externally validated diagnostic accuracy.

**Table 1:** Comparison of baseline characteristics and levator hiatus parameters between the prolapse and control groups.

Variable	Prolapse group (n = 62)	Control group (n = 60)	*t*-value	*P*-value
Age (years)	33.4 ± 2.9	33.0 ± 2.7	0.80	.428
Height (cm)	160.2 ± 3.2	161.0 ± 3.9	-1.19	.237
Weight (kg)	61.4 ± 3.7	57.8 ± 3.7	5.41	<.001
BMI (kg/m^2^)	23.8 ± 1.0	22.3 ± 1.0	8.72	<.001
Levator hiatus area (cm^2^)				
At rest	15.7 ± 2.1	12.2 ± 0.8	12.2	<.001
Valsalva	23.7 ± 2.3	15.9 ± 1.0	23.9	<.001
Area expansion	8.0 ± 1.0	3.7 ± 0.9	25.0	<.001

**Table 2:** Correlation analysis between BMI and levator hiatus parameters.

Variable	Correlation coefficient (ρ)	*P*-value
Resting area	0.484	<.001
Valsalva area	0.611	<.001
Area expansion	0.657	<.001

**Table 3:** Hierarchical regression analysis of factors influencing levator hiatus parameters.

Model	*R* ^2^	Adjusted *R*^2^	Standard error of estimate	Δ*R*^2^	*P*-value of Δ*R*^2^
Resting area					
1	0.552	0.548	1.6124	0.552	<.001
2	0.552	0.545	1.6192	0.000	.988
3	0.552	0.541	1.6260	0.000	.995
4	0.552	0.537	1.6330	0.000	.998
Valsalva area					
1	0.826	0.825	1.8021	0.826	<.001
2	0.830	0.827	1.7914	0.004	.120
3	0.830	0.826	1.7988	0.000	.905
4	0.830	0.824	1.8060	0.000	.802
Area expansion					
1	0.839	0.837	0.9393	0.839	<.001
2	0.850	0.848	0.9082	0.012	.003
3	0.850	0.847	0.9118	0.000	.804
4	0.851	0.846	0.9147	0.000	.623

**Table 4:** Multiple linear regression analysis of factors influencing levator hiatus area expansion.

Variable	Unstandardized Coefficients (B)	Standard Error	Standardized Coefficients (Beta)	t-value	*P*-value
Constant	−1.770	1.798	–	−0.985	.327
Group	3.847	0.210	0.829	18.305	<.001
BMI	0.246	0.080	0.139	3.059	.003

**Table 5:** Diagnostic performance of levator hiatus parameters for bladder prolapse.

Indicator type	Indicator	AUC (95% CI)	Optimal Cutoff	Sensitivity (%)	Specificity (%)	*P*-value
Static morphological	Resting area	0.920 (0.862–0.978)	>13.25 cm^2^	87.1	93.2	<.001
	Valsalva area	0.999 (0.997–1.000)	>18.15 cm^2^	96.8	98.3	<.001
Dynamic functional	Area expansion	1.000 (1.000–1.000)	>5.85 cm^2^	100.0	100.0	<.001

**Figure 1.**
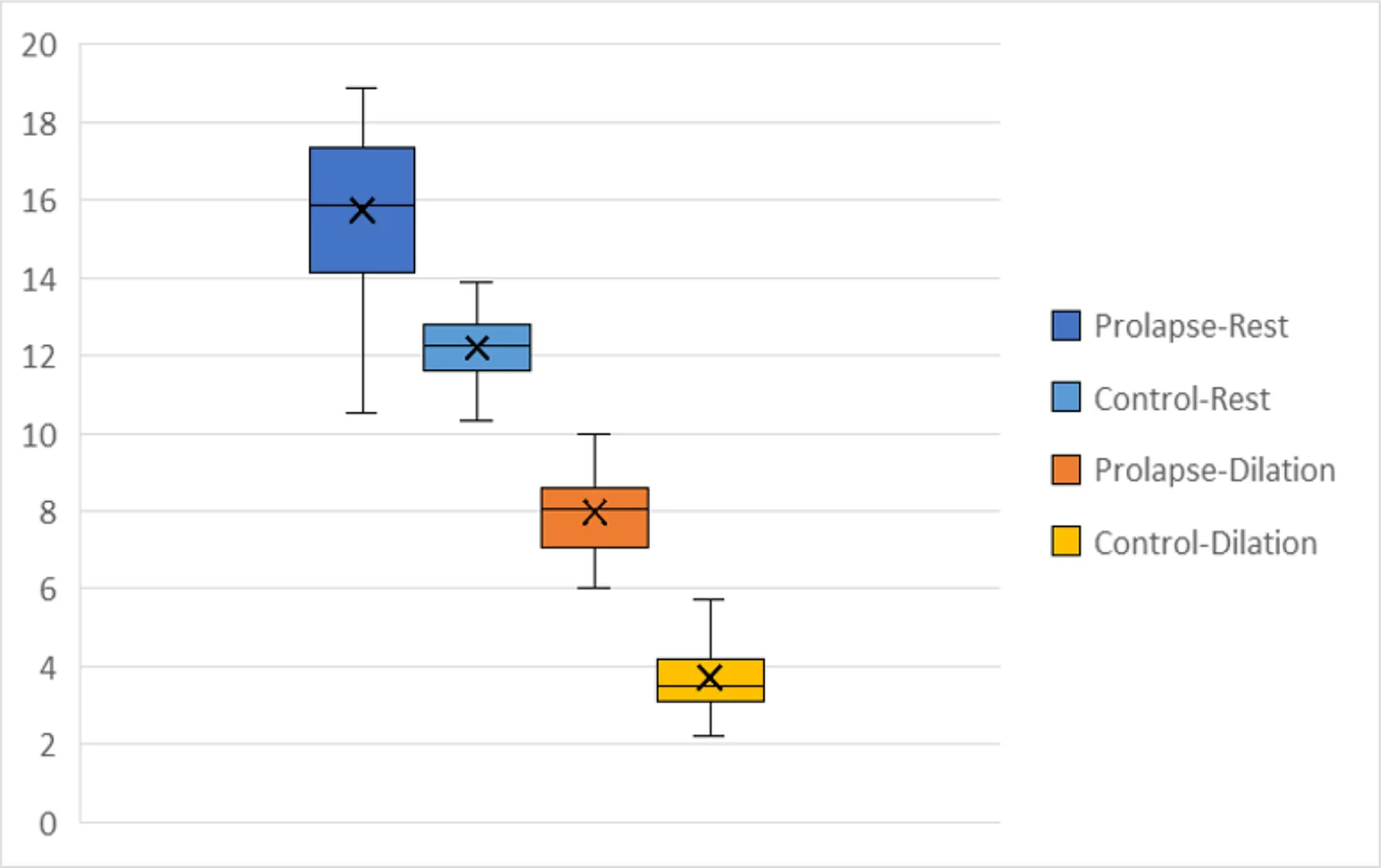
Distribution of static and dynamic indicators of the levator hiatus in the prolapse and control groups.

**Figure 2.**
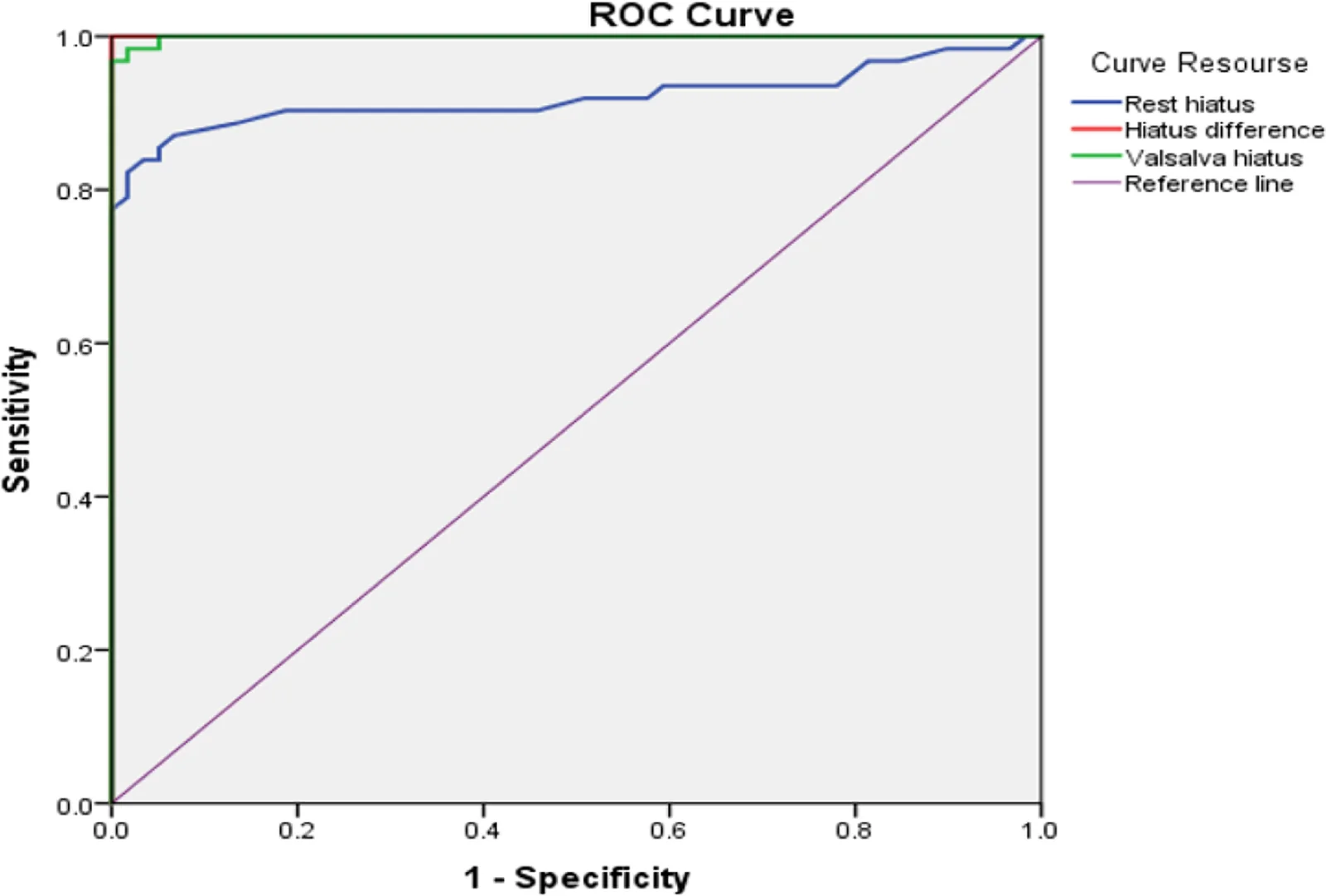
ROC curves of levator hiatus parameters for diagnosing pelvic organ prolapse.

## 4. Discussion

### 4.1. Pregnancy, childbirth, and bladder prolapse

Perineal trauma during childbirth can be associated with anatomical injury and functional remodeling of the levator ani and surrounding tissues, and postpartum women may subsequently develop PFD such as bladder prolapse.^[2]^ Studies by Sascha et al^[3]^ have shown that the incidence of bladder prolapse is higher in vaginally parous women compared with nulliparous women, with a higher proportion of type III prolapse. Previous studies have also documented postpartum changes in pelvic floor strength and geometry. In the present study, the use of nulliparous controls provides a low-exposure reference for childbirth-related remodeling, but it also means that parity differs systematically between the prolapse and control groups. Consequently, the observed between-group differences cannot be attributed specifically to bladder prolapse; they may also partly reflect pregnancy- and childbirth-related changes. This design feature should be considered when interpreting all group comparisons.

### 4.2. Bladder prolapse and structural changes in the levator hiatus

The levator ani muscle surrounds the urogenital hiatus, and its strength and integrity are crucial for maintaining the structure and size of the hiatus and supporting pelvic organs. Research indicates that pelvic organ prolapse and its progression are strongly correlated with levator hiatus expansion, which is an independent risk factor for POP occurring 4 to 10 years postpartum.^[4]^

Previous studies have shown reduced pelvic muscle strength postpartum,^[5,6]^ increased levator hiatus area,^[7]^ increased symptoms of POP,^[8]^ and a lower bladder neck position in parous women compared to nulliparous women.^[9,10]^ Women with bladder prolapse exhibit significantly larger levator hiatus areas than healthy women, with hiatus expansion during maximum Valsalva maneuver reaching 30 and 11% in the 2 groups, respectively.^[11,12]^ This study found that the levator hiatus was significantly larger at rest and during maximum Valsalva maneuver in the prolapse group, consistent with previous findings.

### 4.3. Bladder prolapse and functional changes in the levator ani

Most previous research has focused on static anatomical measurements, with limited understanding of dynamic pelvic floor function, particularly the change in levator hiatus area between rest and Valsalva. By comparing resting area, Valsalva area, and area expansion, this study identified what we describe as a statistical “discordance”: static measures and dynamic area expansion showed different association patterns with BMI in the fitted models. Here, “discordance” refers only to this difference in statistical association patterns and should not be interpreted as evidence that morphology and function arise from separate causal pathways. Alternative explanations include parity-related differences between groups, residual confounding, within- and between-operator measurement variability, differences in Valsalva effort, and sampling variability.

The significant differences in static morphological indicators between the prolapse and control groups persisted after inclusion of BMI in the regression models; group status explained a large proportion of the variance, and BMI was not a significant additional predictor. These results suggest that BMI did not account for the observed group differences within this dataset. However, because parity was not balanced or independently modeled, and other potential confounders were not available, these findings should not be interpreted as proving that the group differences are attributable to prolapse itself or are independent of childbirth-related effects.

For levator hiatus area expansion, BMI showed a statistically significant linear association after adjustment for group status (B = 0.246, standardized β = 0.139, *P* = .003), and the association was similar across the fitted groups because no significant interaction was identified. This finding indicates an association between higher BMI and greater rest-to-Valsalva area change in this sample. Possible explanations include differences in intra-abdominal loading or tissue mechanical properties, but these mechanisms were not measured directly and cannot be established from the present cross-sectional data. Measurement variability, differences in Valsalva performance, parity-related pelvic floor changes, and unmeasured connective-tissue or obstetric factors may also contribute to the observed association.

The dynamic area-expansion measure showed higher apparent diagnostic performance than the static morphological measurements in this dataset. At the data-derived cutoff value of 5.85 cm^2^, sensitivity and specificity were both 100%, while the Valsalva and resting areas had AUCs of 0.999 and 0.920, respectively. These estimates should be interpreted cautiously. The ROC analysis was performed in the same cohort used to derive the cutoff, the sample was a case-control sample with markedly different parity status between groups, and no internal resampling or external validation cohort was available. Accordingly, the observed AUC of 1.000 may overestimate performance in independent clinical populations. The result is best viewed as hypothesis-generating evidence that dynamic area change may provide complementary discriminatory information, requiring validation in parity-matched and prospectively recruited cohorts.

This study has several important limitations. First, the cross-sectional case-control design precludes causal inference; observed associations cannot establish whether altered levator hiatus dynamics precede, result from, or merely coexist with bladder prolapse. Second, parity status differed completely between groups: all cases were primiparous and all controls were nulliparous. Therefore, pregnancy- and childbirth-related remodeling is inseparable from case-control status, and the findings cannot distinguish prolapse-related differences from parity-related differences. Third, ROC thresholds and performance estimates were derived and evaluated in the same relatively small cohort, which may lead to optimistic estimates, particularly the AUC of 1.000; external validation is required. Fourth, formal intra-observer and inter-observer reliability analyses for levator hiatus measurements were not available, and operator-specific identifiers and blinding status were not documented, so measurement and observer variability cannot be excluded. Fifth, adjustment for confounding was limited primarily to BMI. Potentially relevant factors such as mode of delivery, time since delivery, neonatal birth weight, levator ani injury, connective-tissue characteristics, age-related tissue changes, and standardized measures of Valsalva effort were not comprehensively modeled. Finally, the use of a single-center sample may limit generalizability. Future studies should include parity-matched control groups, standardized and blinded ultrasound acquisition/analysis, formal reproducibility testing, broader confounder assessment, and prospective external validation.

In summary, the present statistical analyses show different association patterns for static levator hiatus morphology and dynamic area expansion. Static measures were strongly associated with case-control group status, whereas area expansion was additionally associated with BMI. These findings are compatible with the possibility that dynamic assessment captures information not fully represented by static measurements, but they do not establish distinct pathophysiological mechanisms. Clinical use of rest-to-Valsalva levator hiatus area expansion should therefore be considered investigational until its reproducibility and diagnostic performance are confirmed in appropriately matched and independent populations.

## Author contributions

**Conceptualization:** Yuhong Xue, Liang Mu, Shuliang Nan.

**Data curation:** Yuhong Xue, Liang Mu, Shuliang Nan.

**Formal analysis:** Yuhong Xue, Liang Mu, Shuliang Nan.

**Funding acquisition:** Shuliang Nan.

**Investigation:** Shuliang Nan.

**Writing – original draft:** Shuliang Nan.

**Writing – review & editing:** Shuliang Nan.
